# Effect of densification on the physical and mechanical properties of the inner part of oil palm trunk impregnated with methylene diphenyl diisocyanate

**DOI:** 10.1038/s41598-022-19504-x

**Published:** 2022-09-12

**Authors:** Silvia Uthari Nuzaverra Mayang Mangurai, Dede Hermawan, Yusuf Sudo Hadi, Ignasia Maria Sulastiningsih, Efrida Basri, Imam Busyra Abdillah, Muhammad Iqbal Maulana, Byantara Darsan Purusatama, Se Yeong Park, Seung Hwan Lee, Fauzi Febrianto, Nam Hun Kim

**Affiliations:** 1 Department of Forest Products, Faculty of Forestry and Environment, IPB University (Bogor Agricultural University), Bogor, 16680 Indonesia; 2 Research Center for Biomass and Bioproducts, National Research and Innovation Agency (BRIN), Bogor, 16911 Indonesia; 3 Institute of Forest Science, Kangwon National University, Chuncheon, 24341 Republic of Korea; 4 Department of Forest Biomaterials Engineering, College of Forest and Environmental Sciences, Kangwon National University, Chuncheon, 24341 Republic of Korea; 5 Faculty of Forestry, University of Tanjungpura, Pontianak, West Kalimantan 78124 Indonesia

**Keywords:** Engineering, Biomaterials, Composites

## Abstract

Oil palm (*Elaeis guineensis* Jacq.) plantations in Indonesia are increasing over the past few years. After economic productivity, however, the unproductive oil palm trunks are felled and mostly go to waste, especially the inner part of the oil palm trunk (IOPT). There are several modification methods to utilize IOPT, such as impregnation and densification. Methylene diphenyl diisocyanate (MDI) is a common resin used for impregnation in composite industries because it is non-toxic and has excellent physical and mechanical properties but it has never been applied for the impregnation of IOPT. This study aimed to analyze the effect of densification on the physical and mechanical properties of the inner part of oil palm trunk (IOPT) impregnated using methylene diphenyl diisocyanate (MDI) resin to obtain valuable information regarding the efficient utilization of unproductive oil palm trunks. IOPT was densified and compregnated with compression ratios (CRs) of 20% and 30%. The physical properties (density, moisture content (MC), and water absorption (WA)) and mechanical properties (modulus of elasticity (MOE), modulus of rupture (MOR), and hardness) of the compregnated samples were better than those of the densified samples. The density and mechanical properties at CR 30% were higher than those at CR 20%. The improvements in density, MC, and WA of the compregnated IOPT with CR 30% were 127%, 54%, and 70%, respectively, compared to that in untreated IOPT. Furthermore, improvements in the MOE, MOR, and hardness of the compregnated IOPT with CR 30% were 489%, 379%, and 393%, respectively. The mechanical properties of the compregnated IOPT at CR 20% and 30% increased two- to three-fold from strength class V in control IOPT to strength class III in compregnated IOPT with CR 20% and to strength class II in compregnated IOPT with CR 30%, respectively.

## Introduction

Oil palm (*Elaeis guineensis* Jacq.) is the primary estate crop commodity in Indonesia, and plantations have gradually increased over the past few years. The plantation area increased from 14.33 million hectares in 2018 to 14.86 million hectares in 2020^1^. The economic life span of oil palm is 25–30 years, and after economic productivity, the unproductive oil palms are felled and mostly go to waste. Prabuningrum et al*.* ^2^ reported that the rejuvenation phase in Indonesian oil palm plantations produces 220 m^3^ ha^−1^ of unproductive oil palm trunks (OPTs) every year. In addition, Hartono et al.^3^ mentioned that in Indonesia, oil palm plantations produce sawn timber of 50.1 m^3^ ha^−1^ from the hard outer part of the OPT every year, whereas the inner part of the trunk (IOPT) becomes waste.

The OPT consists of vascular bundles and parenchyma, and there are variations in the proportion of vascular bundles and parenchyma between the inner and outer parts. Bakar et al.^4^ reported that juvenile vascular bundles are dominant in IOPT, whereas mature vascular bundles are dominant in outer oil palm trunks (OOPTs). Moreover, the number of parenchyma tissues increased from the outer to inner parts. OOPT had a higher density than IOPT, which was caused by the uneven proportion of vascular bundles and parenchyma tissues along the radial direction of the OPT. Hartono et al.^5^ stated that the density of OPT ranged from 0.23 g cm^−3^ for the inner part to 0.74 g cm^−3^ for the outer part. Bakar et al.^6^ also reported that the IOPT density ranged from 0.30 to 0.40 gcm^−3^. In general, OOPT is only one-third of the trunk diameter, whereas IOPT is two-thirds of the OPT diameter^7^. However, IOPT is not commonly used by the industry because of its low density, dimensional stability, strength, durability, and machining. Therefore, to maximize the potential of IOPT, modification is required to improve its undesirable properties.

Several studies have reported that the undesirable physical and mechanical properties of IOPT can be improved by modification methods such as impregnation, densification, and compregnation (impregnation followed by densification). The mechanical properties of the impregnated IOPT modified with low molecular weight phenol formaldehyde (LmwPF) were better than those of untreated IOPT. However, the mechanical properties of the impregnated IOPT were inferior to those of the impregnated OOPT^8^. Dungani et al.^9^ also reported that OPT impregnated with a mixture of phenol formaldehyde (PF) resin and nanoparticles of oil palm shell showed higher density and water absorption (WA) improvement than OPT impregnated with PF only. Prabuningrum et al.^2^ showed that laminated boards prepared from densified IOPT had lower modulus of elasticity (MOE) and modulus of rupture (MOR) than those prepared from OOPT. Choowang and Suklueng^10^ mentioned that densification at 140 °C increased the density of IOPT from 0.40 to 0.43 g cm^−3^. Furthermore, as mentioned by Srivaro et al.^11^, the densification process on low density coconut wood increased the bending strength, modulus of elasticity, compressive strength parallel to grain, and perpendicular-to-grain shear strength of the wood up to 125%, 54%, 112%, and 129%, respectively. The IOPT compregnated with PF improved its density, MOE, and MOR by 135%, 251%, and 275%, respectively^3^. Furthermore, Aizat et al.^12^ reported that OPT compregnated with PF decreased WA by 288% and improved MOE and MOR by 366% and 433%, respectively. Compregnation of IOPT using tannin resorcinol formaldehyde showed an increase in hardness value about 70%^13^. Based on previous studies, it is clear that the physical and mechanical properties of IOPT compregnated with PF resin were better than those of impregnated or densified IOPT. However, the reagents used for formaldehyde-based impregnation emit volatile organic compounds that are harmful to human health. Therefore, environmentally friendly and human-friendly resins, such as methylene diphenyl diisocyanate (MDI), are required.

Methylene diphenyl diisocyanate (MDI) is a common resin used in wood composite industries because it is non-toxic and has excellent physical and mechanical properties. MDI has a low molecular weight of 364 g mol^−1^ and can easily penetrate OPT^14,15^. Ling et al.^16^ and Papadopoulos^17^ reported that particleboards bonded with MDI had better bending strength and internal bond strength than particleboards bonded with urea–formaldehyde (UF), melamine–formaldehyde (MF), and PF. Ling et al*.*^16^ mentioned that the MOE, MOR, and internal bonding of straw particleboard increased with an increasing amount of applied MDI. The authors also reported that the MDI molecule has two –N=C=O; one can expect a response with the –OH on the surface of straw particles by hot-press molding, and the other can have the same response with the adjacent straw particle surface, which can form a solid bonding in straw particles. However, MDI as an agent for impregnation of IOPT has never been reported, except for Mangurai et al.^18^. As the authors reported, IOPT impregnated and compregnated with 20% MDI increased the density and its resistance to subterranean termites.

As mentioned above, IOPT has potency as alternative raw material for the wood industry, and there are modification methods, such as impregnation, densification, and compregnation, that can improve the undesirable properties of IOPT. PF-based resin is commonly used as an agent for the impregnation of IOPT, which is harmful to human health. On the other hand, MDI is an environmentally friendly and human-friendly resin commonly used in wood composite industries and has excellent physical and mechanical properties. Our previous study reported that IOPT impregnated with MDI with a concentration of 20% were proven to increase the density and its resistance to subterranean termites^18^. However, to date, there is no study on the physical and mechanical properties of impregnated and compregnated IOPT using MDI as an agent for the impregnation. Therefore, this study aimed to analyze the effect of densification on the physical and mechanical properties of IOPT impregnated with MDI resin to provide valuable information regarding the efficient utilization of IOPT.

## Materials and methods

### Materials

**Figure 1:**
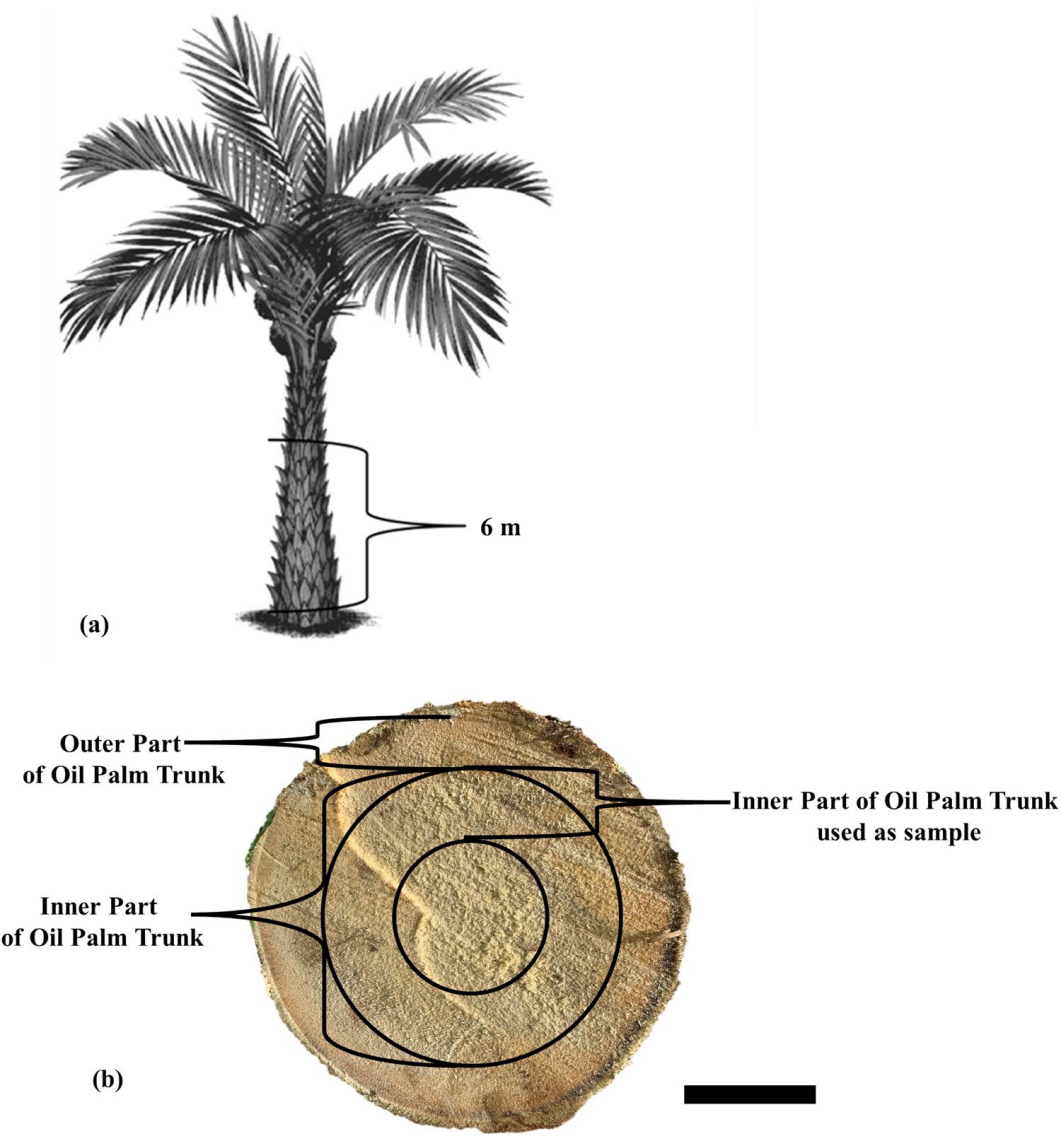
Illustration of sampling of inner part of oil palm trunk (IOPT). (**a**) Partition of axial direction. (**b**) Black circled area in cross section indicates the IOPT used as a sample in this study. Scale bar: 100 mm.

Four unproductive OPTs aged 35 years were obtained from an oil palm plantation on the IPB University (Bogor Agricultural University) campus, Bogor, West Java, Indonesia. The OPT samples were obtained in compliance with all institutional, national, and international guidelines and legislation. Basic information on the harvested oil palms is presented in Table 1. The IOPT used was one-third of the middle part of the trunk diameter, as shown in Fig. 1. The MDI used contained 40–44% resin with a viscosity of 5000–15,000 cps and a pH of 6.5–8.5 and was purchased from Polychemie Asia Pacific Ltd., Jakarta, Indonesia.

**Table 1:** Basic information of unproductive oil palm trunks used in this experiment.

Sample	Number	Age	length (m)	Diameter (cm)	Location
OPT	4	35 years (from 1985 to 2020)	6.9 ± 0.4	30.6 ± 1.0	Bogor, Indonesia (6°33′5.55″S, 106°42′55.35″E)

### Methods

#### Sample preparation

The IOPT was air-dried using a fan to prevent fungal infection. Air-dried IOPT with a moisture content (MC) of 14–16% was cut to obtain the samples. The modification code Samples with dimensions of 15 (R) × 50 (T) × 50 (L) mm^3^ were used for the evaluation of physical properties and hardness, and those with dimensions of 15 (R) × 25 (T) × 410 (L) mm^3^ were used for the bending property test, as shown in Table 2.

**Table 2:** Sample information for evaluating the physical and mechanical properties of modified IOPT.

Experiments	Sample size	Modification code	Number of specimens	Total
Weight percent gain (WPG)	15 (R) × 50 (T) × 50 (L) mm^3^	I, ID20, and ID 30	5 × 3	15
Compression set (C-set)		D20, D30, ID20, and ID30	5 × 4	20
Recovery set (RS)		D20, D30, ID20, and ID30	5 × 4	20
Density		U, I, D20, D30, ID20, and ID30	5 × 6	30
Moisture content (MC)		U, I, D20, D30, ID20, and ID30	5 × 6	30
Water absorption (WA)		U, I, D20, D30, ID20, and ID30	5 × 6	30
MOE and MOR	15 (R) × 25 (T) × 410 (L) mm^3^	U, I, D20, D30, ID20, and ID30	5 × 6	30
Hardness	15 (R) × 50 (T) × 50 (L) mm^3^	U, I, D20, D30, ID20, and ID30	5 × 6	30

*R: Radial direction; T: Tangential direction; L: Longitudinal direction; U = Untreated; I = Impregnation; D20 and D30 = Densification with compression ratio (CR) 20% and 30%, respectively; ID20 and ID30 = compregnation with CR 20% and 30%, respectively.

#### Impregnation and densification

**Figure 2:**
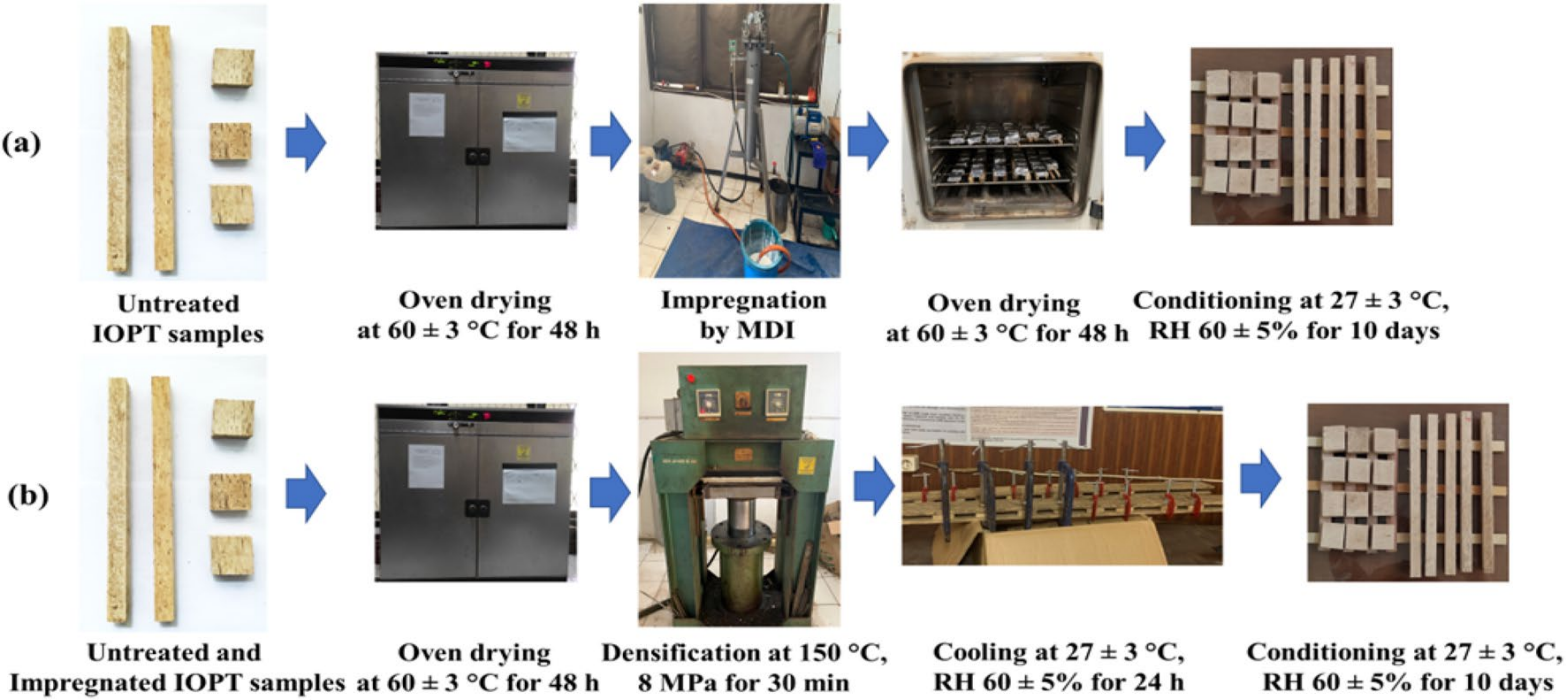
Modification process of the inner part of oil palm trunk. (**a**) Impregnation process of untreated IOPT samples; (**b**) Densification process of untreated and impregnated IOPT samples.

A flowchart of the modification process is shown in Fig. 2. The samples with dimension of 15 (R) × 50 (T) × 50 (L) mm^3^ and 15 (R) × 25 (T) × 410 (L) mm^3^ were oven-dried at 60 ± 3 °C for 48 h, and then the weights and volumes of the samples were measured prior to impregnation. IOPT impregnation was performed with 20% MDI. Next, the samples were loaded into a 7-L vacuum-pressure vessel and vacuumed under 7.6 MPa for 15 min. After releasing the vacuum, the MDI resin was pumped into the vessel until it reached a pressure of 1.2 MPa, and this condition was maintained for 1 h^19^. Next, the sample was drained, wrapped with aluminum foil, and oven-dried at 60 ± 3 °C until a constant weight was achieved. Finally, the MDI-impregnated samples were conditioned in a laboratory at 27.5 ± 3 °C and RH 60 ± 5% for ten days before the measurement and compregnation process (impregnation followed by densification).

For densification, the untreated samples were oven-dried at 60 ± 3 °C for 48 h and then densified using a hot-press (06-SAAJS-2000, LIPI, Bogor, Indonesia) at 150 °C under a pressure of 8 MPa for 30 min^20^. The compregnation process was similar to the densification process, but the initial samples were impregnated with 20% MDI. Two different compression ratios (CRs) of 20% and 30% were applied for densification in this experiment. The CR is the ratio of the reduced dimension to the original dimension^21^. A CR of 20–30% is a reasonable condition for improving mechanical properties^22^. The densified and compregnated samples were clamped at 27.5 ± 3 °C and RH 60 ± 5% for 24 h to release heat and prevent springback. After 24 h, the clamps were released and the samples were then conditioned in a laboratory at 27.5 ± 3 °C and RH 60 ± 5% for 10 days.

#### Measurement of physical properties

Weight percent gain (WPG) is the sum of sample weight after impregnation and compregnation relative to the oven-dried weight of the wood. The WPG of impregnated and compregnated IOPT was measured from five samples of each modification process, which were oven-dried at 60 ± 3 °C for 48 h. The oven-dried weight was measured before and after treatment. The WPG was calculated using Eq. (1).

1 $$WPG\left( \% \right) = \frac{{W_{2} - W_{1} }}{{W_{1} }} \times 100$$

where W_1_ and W_2_ are the oven-dried weights of the IOPT samples before and after the treatment, respectively.

The degree of compression in the densified and compregnated IOPT was determined using the compression set (C-set). The recovery set (RS) is the degree of recovery relative to the original size of the wood after densification owing to the inner stress in the wood^23,24^. Five samples from each densified and compregnated IOPT samples at CRs of 20% and 30% were used to measure the C-set. For the RS, five samples each from densified and compregnated IOPT at CR 20% and 30% were oven-dried at 103 °C for 24 h. Subsequently, the samples were immersed in room temperature water for 24 h and oven-dried again at 103 °C for 24 h. This cycle was performed three times. The C-set and RS values were calculated using Eqs. (2) and (3), respectively:

2 $$\text{C-Set} \left( \% \right) = \frac{{T_{0} - T_{1} }}{{T_{0} }}~ \times 100$$

3 $${\text{RS}} \left( \% \right) = \frac{{T_{2} - T_{1} }}{{T_{0 } - T_{1} }} \times 100$$

where T_0_ is the thickness of the sample dried in the oven at 60 ± 3 °C for 48 h before pressing, T_1_ is the thickness after conditioning at room temperature, and T_2_ is the final thickness of the sample after the immersion cycle.

Five samples each from untreated and modified IOPT with dimensions of 15 (R) × 50 (T) × 50 (L) mm^3^ were used to determine the density and MC of all samples determined according to ASTM D143-94 ^25^. Water absorption (WA) was determined from the weight of the samples before and after immersion^26^. Five samples with dimensions of 15 (R) × 50 (T) × 50 (L) mm^3^ were used to determine the WA of each modified IOPT. The excess water on the samples was wiped, and the weight was measured. WA was calculated using Eq. (4):

4 $$WA \left( \% \right) = \frac{{W_{2} - W_{1} }}{{W_{1} }} \times 100$$

where W_1_ and W_2_ represent the weights of the samples before and after immersion for 24 h, respectively.

#### Measurement of mechanical properties

The mechanical properties (MOE, MOR, and hardness) were tested based on the ASTM D143-94^25^ standard using a universal testing machine (CV-6040A4, Chun Yen Co., Ltd., Taichung, Taiwan). The MOE and MOR of the untreated and modified IOPTs were measured from five 15 (R) × 25 (T) × 410 (L) mm^3^ samples. A single-point loading was applied on the tangential surface of the samples with a crosshead speed of 3.5 mm/min. The untreated and modified IOPT were classified into a strength classification of Indonesian wood based on the average value of MOR, as Martawijaya et al.^27^ and shown in Table 3.

**Table 3:** Strength classification of Indonesian wood with bending strength^27^.

Wood strength class	Modulus of rupture (MPa)
I	> 108
II	71–108
III	49–71
IV	35–49
V	< 35

Hardness was determined using the Janka test from five samples of 15 (R) × 50 (T) × 50 (L) mm^3^. A hemispherical steel ball with a 1.128 + 0.005 cm diameter was loaded onto the tangential surface of the samples. The load was measured at a depth of 0.564 cm.

#### Scanning electron microscopy (SEM)

The size of the samples used for microscopy was 5 (R) × 5 (T) × 5 (L) mm^3^. The samples were placed on the carbon tape attached to a holder with a diameter of 10 mm. The morphology of the transverse surface of the untreated and modified IOPT was observed using a field emission scanning electron microscope (FE-SEM, Thermo Scientific Quattro S, Brno, Czech Republic) at an accelerating voltage of 10 kV.

#### Data analysis

Data were analyzed using Microsoft Excel 2019 and SPSS Statistics version 22. Data on the physical and mechanical properties were analyzed using a completely randomized design. Six levels of CR (untreated, impregnated, densified CR 20%, densified CR 30%, compregnated CR 20%, and compregnated CR 30%) were tested. Data were also analyzed using Duncan’s multiple range test to obtain the differences between treatments at 5% level of significance.

## Results and discussion

### Physical properties

#### WPG

The WPGs of the samples impregnated and compregnated with 20% and 30% CR are shown in Table 4. The WPG of impregnated IOPT was 28.2%, whereas that of compregnated IOPT with CR 20% and 30% was 15.0% and 12.7%, respectively. Duncan’s multiple range test showed that the WPG value of impregnated IOPT was significantly higher than that of compregnated IOPT. Regarding the difference in WPG value between modification methods, Aizat et al.^12^ stated that the resin was not fully cured during the drying stage in the impregnation process, and the resin was squeezed out during the densification process. The WPG of the compregnated IOPT with CR 30% was lower than that of the compregnated IOPT with CR 20%. Therefore, a higher CR results in a higher amount of resin squeezing out from the sample ^12^. However, there was no significant difference in the WPG between compregnated IOPTs at 20% CR and those at 30% CR.

**Table 4:** Weight percent gain, compression set, and recovery set values of modified inner part of oil palm trunk.

Modification	Weight percent gain (%)	Compression set (%)	Recovery set (%)
U	–	–	–
I	28.2 (3.3) b	–	–
D20	–	17.4 (3.0) a	73.3 (14.7) c
D30	–	28.3 (5.5) b	63.4 (19.1) bc
ID20	15.0 (3.6) a	18.3 (1.4) a	48.8 (7.0) ab
ID30	12.7 (2.14) a	35.7 (1.8) c	39.5 (13.8) a

Notes: Values in parentheses are standard deviations. U = Untreated; I = Impregnation; D20 and D30 = densification with compression ratio (CR) 20% and 30%, respectively; ID20 and ID 30 = compregnation with CR 20% and 30%, respectively. Different letters in the same column indicate significant differences at 5% level of significance, based on Duncan’s multiple range test.

#### C-set and RS

The C-set and RS values of the densified and compregnated IOPT samples are listed in Table 4. The C-set value of densified IOPT was 17.4% for a CR of 20% and 28.3% for a CR of 30%. In addition, the C-set values of the compregnated IOPT with CRs of 20% and 30% were 18.3% and 35.7%, respectively. In densified and compregnated IOPT, the C-set value increased with increasing CR. In addition, the samples compregnated with CR 30% yielded significantly higher C-set values than those densified with CR 30%, whereas there was no significant difference in C-set values between densified OPT at CR 20% and compregnated IOPT at CR 20%. The higher C-set value at CR 30% is presumably caused by a higher cavity compaction caused by 30% CR than 20% CR. Ashaari et al.^28^ reported that cavity volume reduction of compregnated Jelutong wood with LmwPF resin increased with increasing CR during the densification process. Therefore, a higher CR results in a denser structure of woody materials, as shown in Table 5. Furthermore, the higher C-set values of compregnated IOPT compared to that of densified IOPT could be due to the MDI filling in the cavity of IOPT. Lykidis et al.^29^ stated that the C-set value of compregnated poplar wood with MF resin was higher than that of densified wood. The authors also explained that the compregnated wood formed new bonds between the cell wall components and the resin, resulting in a denser wood structure.

**Table 5:** Density, moisture content, and water absorption of modified inner part of oil palm trunk.

Modification	Density (g cm^-3^)	Improvement in density (%)	Moisture content (MC, %)	Reduction in MC (%)	Water absorption (WA, %)	Reduction in WA (%)
U	0.30 (0.03) a		17.80 (0.31) d		112.71 (22.35) d	
I	0.41 (0.03) ab	+ 37	11.62 (0.13) b	− 35	83.48 (11.32) c	− 26
D20	0.48 (0.06) bc	+ 60	12.77 (0.66) c	− 28	69.84 (10.03) bc	− 38
D30	0.56 (0.12) cd	+ 87	12.13 (0.63) b	− 32	58.12 (14.81) b	− 48
ID20	0.58 (0.03) d	+ 93	7.96 (0.46) a	− 55	29.59 (4.17) a	− 74
ID30	0.68 (0.08) e	+ 127	8.25 (0.52) a	− 54	33.42 (10.07) a	− 70

Notes: Values in parentheses are standard deviations. U = Untreated; I = Impregnation; D20 and D30 = densification with compression ratio (CR) 20% and 30%, respectively; ID20 and ID 30 = compregnation with CR 20% and 30%, respectively. Different letters in the same column indicate significant differences at the 5% level of significance based on Duncan’s multiple range test.

The RS values of the densified and compregnated IOPT were 63.4–73.3% and 39.5–48.8%, respectively. The recovery set of the densified IOPT was significantly higher than that of the compregnated IOPT, and the RS values decreased significantly with increasing CR. The lower RS values in the compregnated IOPT were presumably due to MDI filling in the cavity of the IOPT structure and the resin restraining springback after the densification process. Fang et al.^30^ stated that the porous structure of densified aspen wood veneer was recovered by water immersion due to the reopening of the deformed cavity. Densified wood without resin facilitates WA and releases more internal stress^31^. In addition, Bao et al.^32^ stated that a significant reduction in the RS of thermo-hydro-mechanical densified wood with a high CR could be explained by a breakdown of the cross-linkage between the cell wall components, which is responsible for the memory effect in wood.

#### Density, moisture content (MC), and water absorption (WA)

The density, MC, and WA of the modified IOPT are listed in Table 5. The density of untreated and impregnated IOPT was 0.30 g cm^−3^ and 0.41 g cm^−3^, respectively, and that of densified IOPT was 0.48 g cm^−3^ at CR 20% and 0.56 g cm^−3^ at CR 30%. The density of compregnated IOPT at CR 20% and 30% was 0.58 and 0.68 g cm^−3^, respectively. Compared to that of untreated IOPT, the density of IOPT increased by 37% with impregnation, whereas the increase was 60% and 80% with densification at 20% and 30% CR, respectively. Moreover, compregnation at CR 20% and 30% increased the density by 93% and 127%, respectively. The modification process significantly increased the density of the untreated IOPTs, and there were significant differences among the densities of the modified IOPTs. The density of the compregnated IOPT was significantly higher than that of the densified OPT, which could be caused by the combination of impregnation with MDI and densification treatment. As reported by Purba et al*.*^33^, impregnation and densification combinations increased the density of phenol-treated wood. Regarding the mechanism of density increase by impregnation, Malik and Santoso^34^ mentioned that high pressure and temperature maximized the penetration of polymerized Merbau extractive resin into empty cavities of OPT.

The significant density increase with increasing CR must be due to the production of more compacted IOPT with 30% CR than that with 20% CR, which is supported by Ang et al.^35^. The authors reported that the higher compression resulted in a greater decrease in the void volume in the compregnated Mahang wood (*Macaranga sp*.) compared to that in the compregnated wood under low compression, thereby increasing density, rigidity, and hardness.

**Figure 3:**
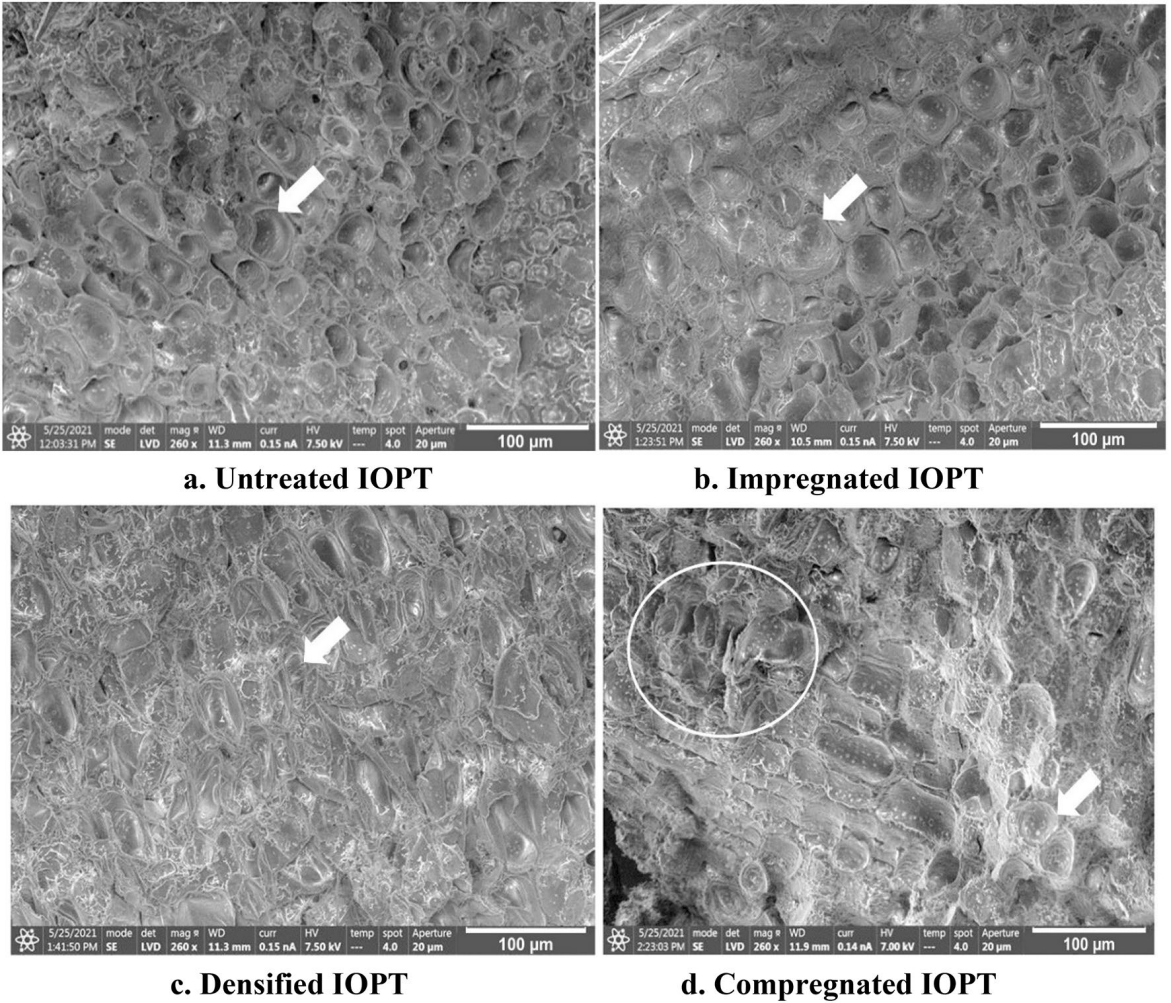
Scanning electron micrographs of the transverse surface in (**a**) untreated palm trunk, (**b**) impregnated inner part of oil palm trunk (IOPT), (**c**) densified IOPT, and (**d**) compregnated IOPT. (**a**) Empty parenchyma lumen in untreated IOPT (arrow); (**b**) methylene diphenyl diisocyanate (MDI)–filled parenchyma lumen in impregnated IOPT (arrow). (**c**) Deformed parenchyma lumen in densified IOPT (arrow). (**d**) Deformed parenchyma lumen (circle) and MDI-filled parenchyma lumen (arrow) in the compregnated IOPT. Scale bars: 100 µm.

Scanning electron micrographs of the transverse surfaces of the untreated, impregnated, densified, and compregnated IOPT are shown in Fig. 3. Untreated IOPT showed an empty parenchyma lumen (Fig. 3a), whereas the lumen of parenchyma cells of impregnated IOPT was filled with MDI (Fig. 3b). Thus, the density of the impregnated IOPT was higher than that of untreated IOPT. In contrast, collapsed parenchymal cells were observed in the densified IOPT (Fig. 3c). This explains why the densified IOPT exhibited a higher density than the impregnated IOPT. In the densified IOPT, the increased density was due to the reduced volume of the cell lumen, showing a decrease in the porosity of IOPT. The increasing density of the compregnated IOPT can be explained by the combination of mechanisms from both the impregnated and densified IOPT. Some MDI-filled cell lumens and deformations were observed in the compregnated IOPT (Fig. 3d).

The MC of untreated, impregnated, densified, and compregnated IOPT was 17.8%, 11.6%, 12.1–12.8%, and 8.0–8.3%, respectively (Table 5). Compared with that of untreated IOPT, the MC of modified IOPT was reduced by 35% with impregnation, 28–32% with densification, and 54–55% with compregnation treatment. The modified IOPT showed a significantly lower MC than the untreated IOPT, and significant differences were observed between the MCs of the modified IOPTs. Densified IOPT showed a higher MC than impregnated IOPT, whereas compregnated IOPT yielded the lowest MC due to the combination of densification and impregnation processes. The heat generated during the densification process could limit the interaction of moisture with wood by reducing OH groups, breaking chains, and hemicellulose degradation^36^. In addition, the resin is crosslinked with cell wall components to reduce moisture interaction^37^.

The WA values of untreated and impregnated IOPT were 112.7% and 83.5%, respectively. Densified and compregnated IOPT showed a WA of 58.1–69.8% and 29.6–33.4%, respectively (Table 5). The WA values of IOPT were reduced by 26% with impregnation, 38–48% with densification, and 70–74% with compregnation treatment. Compregnated IOPT had significantly lower WA values than densified IOPT and impregnated IOPT, whereas the WA of densified IOPT was significantly different from that of impregnated IOPT. Compregnation reduced the permeability of IOPT and blocked water interactions by filling and flattening the IOPT cell. The SEM images of IOPT revealed that the cavity of the densified IOPT was deformed, whereas the cavity of the compregnated IOPT was filled by MDI and flattened (Fig. 2), which could prevent water accessibility. Densification treatment can reduce the number of hydroxyl groups and change the amorphous to crystalline state, resulting in a decrease in the WA of wood ^38^. Moreover, heat and pressure in the densification process for compregnated IOPT results in an infusible, insoluble, and hard network of crosslinked polymers^39^, which reduces water penetration. The compregnated IOPT with MDI in this study had a lower WA than that treated with PF resin at a CR of 55%^12^.

### Mechanical properties of modified IOPT

**Figure 4:**
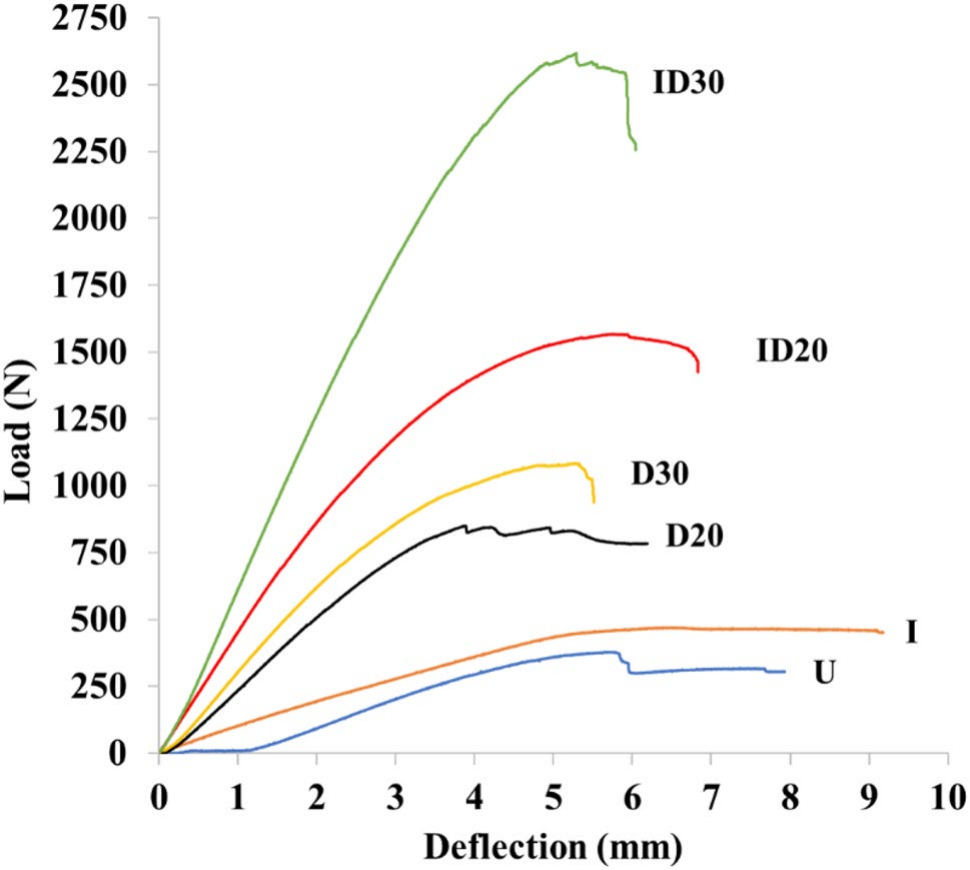
Load–deflection curves of modified inner part of oil palm trunk. U = Untreated; I = Impregnation; D20 and D30 = densification with compression ratio (CR) 20% and 30%, respectively; ID20 and ID30 = compregnation with CR 20% and 30%, respectively.

The load–deflection curves of untreated and modified IOPT are presented in Fig. 4. The compreganted IOPT with CR 20% and 30% showed the highest max load and steeper curve among the modified IOPT and untreated IOPT, which indicates the improvement of bending properties.

The mechanical properties of the modified IOPTs are presented in Table 6. The MOE of the untreated IOPT was 1600 MPa, whereas that of the IOPT impregnated with MDI was 2410 MPa, showing a 51% improvement. The MOE of the densified IOPT was 4474 MPa at a CR of 20% and 5275 MPa at a CR of 30%, which were significantly improved by 180% at CR 20% and 230% at CR 30%. There was no significant difference between the MOEs of densified wood at CR of 20% and 30%. The compregnated IOPT yielded the highest MOE and improvement among the modified IOPT. The MOE of compregnated IOPT was 7636 MPa at a CR of 20% and 9429 MPa at a CR of 30%, which increased by 377% and 489% at a CR of 20% and 30%, respectively. Moreover, the MOE of compregnated IOPT significantly increased with increasing CR. Overall, the modification methods improved the MOE of IOPT, whereas the significant effect of CR only occurred in compregnated IOPT, which could be due to impregnation of MDI resin.

**Table 6:** Mechanical properties of modified inner part of oil palm trunk.

Treatment	MOE (MPa)	Improvement of MOE (%)	MOR (MPa)	Improvement of MOR (%)	Hardness (MPa)	Improvement of hardness (%)
U	1600 (354) a	–	19.61 (7.55) a	–	8.92 (1.96) a	–
I	2410 (457) a	+ 51	25.01 (4.51) ab	+ 28	17.95 (1.37) b	+ 101
D20	4474 (879) b	+ 180	33.83 (9.12) b	+ 73	25.79 (5.98) c	+ 189
D30	5275 (1128) b	+ 230	34.91 (11.67) b	+ 78	26.97 (2.75) c	+ 202
ID20	7636 (1857) c	+ 377	67.47 (13.34) c	+ 244	31.19 (5.49) c	+ 249
ID30	9429 (279) d	+ 489	93.95 (11.08) d	+ 379	44.03 (9.32) d	+ 393

Notes: Values in parentheses are standard deviations. U = Untreated; I = Impregnation; D20 and D30 = densification with compression ratio (CR) 20% and 30%, respectively; ID20 and ID30 = compregnation with CR 20% and 30%, respectively. Different letters in the same column indicate significant differences at 5% level of significance based on Duncan’s multiple range test.

The MOR of the untreated and impregnated IOPT was 19.6 MPa and 25.0 MPa, respectively. The impregnation modification significantly improved the MOR of IOPT by 28%. The MOR of densified IOPT was 33.8 MPa at CR 20% and 34.9 MPa at CR 30%, which were improved by 73% and 78% at a CR of 20% and 30%, respectively. The MOR of IOPT compregnated with CR 20% and 30% was 67.5 MPa and 94.0 MPa, respectively. Compregnated IOPT showed the greatest improvement in MOR as 244% at a CR of 20% and 379% at a CR of 30%. Overall, modified IOPT significantly improved the MOR of IOPT. In addition, the higher CR, the greater MOR in compregnated IOPT, whereas CR showed no significant impact in densified IPOT. Hartono et al.^40^ reported that the MOE and MOR values of IOPT compregnated with PF were 4616 MPa and 49 MPa, respectively, which were lower than those of IOPT compregnated with MDI in this study.

The hardness of untreated and impregnated IOPT was 8.9 MPa and 18.0 MPa, respectively. The hardness of IOPT improved by 101% after impregnation. The hardness of densified IOPT with CR 20% and 30% was 25.8 MPa and 27.0 MPa, respectively, and that of compregnated IOPT with CR 20% and 30% was 31.2 MPa and 44.0 MPa, respectively. The densification improved the hardness of IOPT by 189–202%, whereas the hardness of IOPT compregnated with MDI increased by 249% at a CR of 20% and 393% at a CR of 30%.

The hardness of the modified IOPT was significantly different from that of untreated IOPT. Densified IOPT showed significantly higher hardness than impregnated IOPT, whereas there was no significant difference in hardness among densified IOPTs at CR 20% and 30% and compregnated IOPT at 20%. The hardness of compregnated IOPT at CR of 30% was significantly higher than that at CR of 20%. Hardness is a mechanical property used to assess the suitability of a wood species as a flooring material^41^. The parallel strand lumber of OPT using MDI had a hardness ranging from 8.5 to 31 MPa^42^, which was lower than the hardness of the compregnated IOPT with MDI in this study. Therefore, it is expected that IOPT compregnated with MDI at CRs of 20% and 30% is suitable as flooring material.

In this study, compregnation yielded the highest improvement in MOE, MOR, and hardness among all modification methods tested (Table 6). The mechanical properties significantly increased with increasing CR in compregnated IOPT, which was not observed in densified IOPT. According to the Indonesian wood strength classification by bending test^26^, untreated IOPT, impregnated IOPT, and densified IOPT belong to strength class V, whereas the compregnated IOPT with 20% CR and 30% CR belongs to strength class III and strength class II, respectively. The improvement in the mechanical properties of the compregnated IOPT could be due to the combination of impregnation with MDI and densification treatment. Ling et al*.*^16^ reported that the mechanical properties of low-cost straw particleboard increased with increasing amounts of MDI resin in UF and MDI resin mixtures. The authors explained that MDI molecule has two –N=C=O bonds where each one can expect a response with the –OH on the surface of straw particles by hot-press molding and can form a solid bond with the adjacent straw particle surface. The densification process in the compregnated IOPT reduces the volume of the IOPT cavity, resulting in denser IOPT with higher strength ^11^. MDI filled the cavity of the IOPT, which increased the mechanical properties of the IOPT. Furthermore, higher CR increases wood density by reducing the cavity volume, resulting in higher MOE, MOR, and hardness values compared to that of untreated wood^16,35,43^.

## Conclusions

The modification of IOPT through impregnation, densification, and compregnation significantly improved its physical and mechanical properties. The MDI resin played an important role in improving the properties of the IOPT in the impregnation and compregnation processes. Compregnated IOPT showed a lower WPG than impregnated IOPT and yielded a higher C-set and smaller RS than densified IOPT. The compregnated IOPT showed the highest dimensional stability and greatest improvement in density and mechanical properties among the modified samples. CR variation significantly affected the C-set and density of densified and compregnated IOPT, whereas there was no significant difference in the WPG of the compregnated IOPT between 20 and 30% CR. CR showed no significant effect on the RS, MC, and WA of densified and compregnated IOPT, whereas the mechanical properties of the compregnated IOPT significantly increased with increasing CR. In conclusion, modification through compregnation with MDI provided the best improvement compared to that provided by other treatments. This suggests that the results of this study support the effective utilization of unproductive OPT materials as an alternative raw material in wood industry.

## Data Availability

The datasets generated and analyzed during the current study are not publicly available but are available from the corresponding author on reasonable request.
